# Virtual reality in school-based health promotion: a mixed-methods evaluation of adolescent alcohol, vaping, and other drug use prevention

**DOI:** 10.1093/heapro/daag002

**Published:** 2026-01-27

**Authors:** Chengzi Jiang, Pamela Saleme, Timo Dietrich, James Durl

**Affiliations:** Department of Tourism and Marketing, Griffith University, 170 Kessels Rd, Brisbane, QLD 4111, Australia; Department of Tourism and Marketing, Griffith University, 170 Kessels Rd, Brisbane, QLD 4111, Australia; Department of Tourism and Marketing, Griffith University, 170 Kessels Rd, Brisbane, QLD 4111, Australia; Department of Tourism and Marketing, Griffith University, 170 Kessels Rd, Brisbane, QLD 4111, Australia

**Keywords:** virtual reality, substance prevention, health behaviour, school-based, alcohol and other drugs, vaping, e-cigarettes

## Abstract

School-based health promotion is a key setting for fostering positive youth health behaviours. Digital and immersive technologies offer promising opportunities to engage young people. This study explores a virtual reality (VR) intervention designed to prevent alcohol, vaping, and cannabis use among secondary school students. The intervention allowed students to navigate realistic, branching scenarios simulating peer pressure and substance use, aiming to enhance refusal strategies, critical thinking, and decision-making skills. A mixed-methods evaluation involving 277 students and nine teachers across four Australian schools was conducted. Postintervention surveys assessed engagement, immersion, emotional responses, and skill development, while focus groups and interviews explored participant experiences. Results indicate that students found the VR experience immersive and valuable, particularly for rehearsing peer resistance and evaluating the consequences of risky behaviours. Teachers viewed the intervention as a powerful tool for prompting reflection and discussion and a strong complement to existing health education curricula. Thematic analysis highlighted the importance of realism and interactivity for student engagement. While some technical and content improvements were identified, both students and teachers considered the VR tool effective for enhancing health literacy and behavioural readiness. This study shows that immersive VR can be a scalable, engaging addition to school-based health promotion, improving prevention skills and confidence in managing substance-related situations. As adolescent health behaviours are increasingly shaped by digital environments, immersive interventions such as VR offer a promising avenue for skill building and reflection. Further research should assess long-term impacts, with greater attention to implementation and equity considerations.

## Graphical Abstract

**Graphical Abstract:**
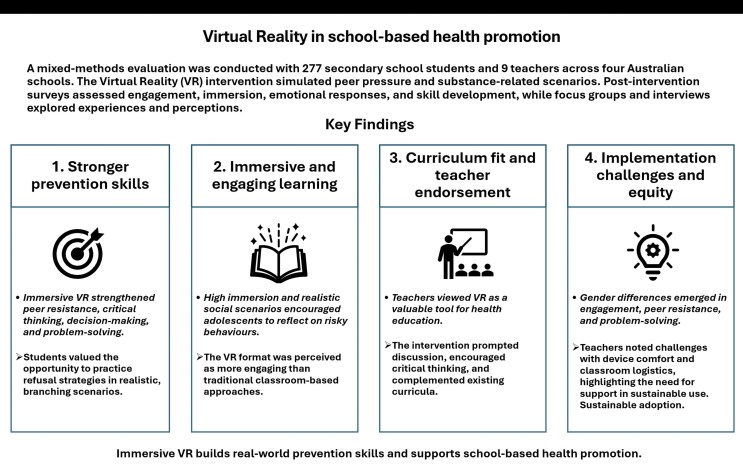

Contribution to Health Promotion

- Demonstrates how immersive virtual reality (VR) can align school-based health promotion with young people’s digital learning preferences.
- Shows that VR can support skill development in peer resistance, decision-making, and critical thinking.
- Provides a scalable model for experiential learning that supports behavioural reflection and real-world application in youth health.
- Offers practical insights into integrating VR into school health education.
- Suggests how immersive tools can enhance motivation and learning in health promotion.

## Introduction

Alcohol, vaping, and other drug (AOD) prevention for youth remains a pivotal public health concern, particularly in countries with high rates of underage substance use. In Australia, underage alcohol consumption declined significantly from the early 2000s to the late 2010s, with a notable increase in the proportion of teenage abstainers. However, since 2019, this trend has plateaued, and rates of underage drinking have begun to rise again. Currently, approximately one-third of Australian adolescents aged 14–17 report consuming alcohol in the past year (Australian Institute of Health and Welfare 2024b). Parallel to this, the use of e-cigarettes among young Australians has increased substantially. In 2023, 9.3% of individuals aged 18–24 reported daily e-cigarette use, highlighting the growing prevalence of vaping among younger demographics (Australian Institute of Health and Welfare 2024a). Emerging nicotine products, such as nicotine pouches, are also gaining popularity among Australian youth, further complicating efforts to address substance use (Jongenelis *et al*. 2024, Watts *et al*. 2024). Compounding these challenges, recent research shows that young people are frequently exposed to online marketing of nicotine products, despite advertising restrictions in many Western countries. Misinformation about health and wellbeing is also increasingly circulated by social media influencers, whose content is often viewed as credible due to high engagement and parasocial relationships. Mulcahy *et al*. (2025) demonstrate that high-virality influencer posts can lower perceived deception and facilitate the spread of misinformation, especially when accompanied by supportive user comments. These dynamics create a digital environment in which adolescents are vulnerable to misleading substance-related content, highlighting the need for forward-looking, media-literate interventions that strengthen critical thinking and digital discernment. McGlinchy *et al*. (2025) similarly found that children as young as 11 frequently encounter vape and tobacco marketing online, where traditional advertising restrictions are often ineffective. Buchanan *et al*. (2018) further show that digital marketing negatively shapes young people’s attitudes and behaviours towards unhealthy products, with peer-endorsed content blurring boundaries between advertising and social interaction. In parallel, adolescents today are growing up in a digital-first environment that strongly influences their health behaviours and perceptions. As Raeside (2025) explains, adolescent health promotion must evolve alongside young people’s digital engagement habits by using community-based and digital-only platforms that reflect their lived experiences and expectations. This involves prioritizing youth voice, digital safety, and participatory design to avoid reinforcing inequities and to address emerging digital determinants of health. In a world-first effort to limit young people’s exposure to harmful online environments, Australia has restricted social media use to individuals aged 16 and over, highlighting growing concern about risks in unregulated digital spaces.

Amid these developments, schools continue to play a central role in universal AOD prevention by providing structured opportunities to shape young people’s attitudes and behaviours before risky substance use patterns emerge. Schools are uniquely positioned for this work because they reach most children and adolescents during key developmental years. The literature shows that social and emotional factors, including peer influence, social norms, and perceived acceptance within family and school environments, are important drivers of adolescent AOD behaviours (Biles *et al*. 2025). The school environment has long been central to public health and educational interventions. Traditional school-based AOD programmes, such as didactic seminars, health education units, and expert-led presentations, aim to delay initiation and reduce substance use by increasing knowledge, shifting attitudes and norms, and enhancing self-efficacy. Yet these approaches often suffer from low engagement, limited personalization, and poor translation of knowledge into practice (Liu *et al*. 2022, Gardner *et al*. 2024). In contrast, emerging approaches such as immersive virtual reality (VR) offer a new vehicle to engage young people through dynamic and experiential learning. VR allows students to actively participate in simulated environments that replicate real-life social scenarios, making abstract concepts more concrete and emotionally resonant (AlGerafi *et al*. 2023, Marougkas *et al*. 2024). By embedding decision-making moments within engaging narratives and real-world 360° footage, VR can support adolescents in critically reflecting on substance use, rehearsing resistance strategies, and building confidence in navigating risky situations. However, despite growing interest, few AOD programmes have integrated or rigorously evaluated VR interventions targeting adolescent substance use, largely due to technological barriers such as cost, equipment requirements, and setup complexity. While VR is known to be engaging (Jiang *et al*. 2026), its potential remains underexplored, as existing studies often rely on limited outcome measures, leaving a critical evidence gap. Building on this knowledge base, this paper examines the implementation of a VR intervention component of a larger AOD programme aimed at high school students. It builds and expands the existing evidence base and explores how VR can influence a range of psychological, emotional, experiential, and behavioural factors such as engagement, immersion, emotional responses, peer resistance, critical thinking, problem-solving, and overall satisfaction. By supporting harm minimization approaches and strengthening practical decision-making and refusal skills, VR offers a promising tool for prevention particularly in the face of growing digital influences on young people’s perceptions and behaviours.

### School-based substance prevention

School settings have long served as a critical platform for delivering preventive health education, including AOD interventions for adolescents (Dai *et al*. 2021, Bold *et al*. 2022, McCauley *et al*. 2023, MacLean *et al*. 2024). As trusted community hubs with daily access to young people, schools are ideally positioned to provide early, universal interventions targeting knowledge, attitudes, and behaviours related to substance use. Public health efforts have leveraged this access to implement structured AOD prevention programmes. However, the effectiveness of such programmes varies considerably depending on design, delivery, and contextual fit. In the specific context of vaping, DiCasmirro *et al*. (2025) identified school-based interventions as one of the primary public health strategies used to address adolescent use. Their scoping review of 38 studies found that interventions were highly variable in content and structure, typically ranging from single-session modules to multi-week programmes. Common content areas included information about the health risks of vaping and the development of refusal skills, with outcome measures focused on knowledge, beliefs, and attitudes. However, the review also noted a lack of consistent behavioural outcomes and limited evidence for long-term effectiveness. Nyman *et al*. (2022) provide complementary insights, highlighting the potential of digital interventions to support refusal self-efficacy, especially when delivered in formats that address the sources of self-efficacy through interactive features. Liu *et al*. (2022) argue that effective e-cigarette prevention requires targeted, youth-centric programming that addresses the specific drivers of adolescent use—including product appeal, misperceptions of harm, and the influence of digital marketing. Their review calls for interventions grounded in empowerment and normative frameworks and emphasizes the importance of rigorous evaluation, particularly through mixed-methods and cluster randomized controlled trials. Gardner *et al*. (2024) provide a comprehensive synthesis showing that while some school-based interventions produce short-term gains in knowledge, attitudes, and intentions, their long-term impact on substance use behaviours is inconsistent and occasionally iatrogenic. Their findings highlight the critical need for schools to implement theory-based, multi-session programmes that are evaluated for real-world effectiveness. Rowland *et al*. (2019) support this view by reviewing Australian school-based AOD programmes and observing that, despite strong evidence for theory-driven approaches such as social influence models, cognitive behavioural therapy, and whole-school frameworks, implementation fidelity and the use of evaluated programmes remain limited. Collectively, these studies highlight growing concern that traditional school interventions may be insufficient for addressing contemporary adolescent substance use, particularly as young people’s social environments and media exposures become increasingly digital (McGlinchy *et al*. 2025, Mulcahy *et al*. 2025). This evolving context calls for new evidence-based approaches that move beyond static content to promote meaningful engagement and behavioural reflection, with immersive VR emerging as one such option.

### Virtual reality in schools

The use of virtual and immersive technologies in education has expanded rapidly in recent years (Radianti *et al*. 2020, Luo *et al*. 2021, Marougkas *et al*. 2024), driven by the shift to remote learning during the coronavirus disease 2019 (COVID-19) pandemic. VR allows learners to interact with complex content in realistic simulated environments, providing multimodal sensory input through visual, auditory, and sometimes haptic channels (Cipresso *et al*. 2018). By cultivating presence and embodied simulation (Riva *et al*. 2018), VR can enhance engagement, comprehension, and knowledge retention. Research has shown that VR serves as a powerful emotional medium capable of eliciting complex emotions, which in turn can enhance memory encoding and support deeper learning (Mancuso *et al*. 2023). Across science, social sciences, health, and medicine, VR has been shown to improve learning of abstract concepts, procedural knowledge, and real-world problem-solving, especially when supported by effective instructional design and scaffolding (Luo *et al*. 2021). Marougkas *et al*. (2024) highlight the importance of personalization in VR education, noting that many earlier applications lacked adaptive content and that gamification and customization are essential for maximizing learning outcomes. Similarly, Khan *et al*. (2023) found that VR’s immersive, game-based design can improve learning outcomes in safety education, offering an engaging and risk-free environment for learners to practice real-world behaviours. Their findings support the broader application of VR in health education settings, where safe skill development and behavioural rehearsal are critical. Marougkas *et al*. (2023) review learning theories relevant to VR and highlight the importance of constructivism, experiential learning, and gamification in designing effective virtual environments, thereby strengthening the theoretical foundation for maximizing VR’s pedagogical value. These attributes make VR particularly appropriate for health education settings that require reflection on personal risk and social behaviour. Mitchell *et al*. (2022) further suggest that reward structures and narrative immersion can enhance autonomy and competency satisfaction in health-focused VR, although relatedness may be harder to achieve in solitary play environments.

### Virtual reality for adolescent alcohol, vaping, and other drug prevention

In the context of adolescent AOD education, VR presents a novel way to simulate complex, high-risk situations young people may encounter around alcohol, vaping, and other drug use (Durl *et al*. 2018). A recent review by Jiang *et al*. (2026) identifies VR-based AOD interventions evaluated across five countries, consistently demonstrating strong engagement and acceptability among adolescents. These studies focused primarily on alcohol prevention (Guldager *et al*. 2020, Hrynyschyn *et al*. 2024), tobacco and vaping (Guo *et al*. 2021, Weser *et al*. 2021), and, in one case, poly-substance use including cannabis (Dietrich *et al*. 2019). Interventions commonly employ immersive, realistic settings to enhance refusal self-efficacy, knowledge, and risk perception. Students consistently report valuing the opportunity to rehearse refusal skills and navigate peer pressure in a safe, simulated environment where emotional resonance and presence enhance engagement and memory (Guldager *et al*. 2020, Lyk *et al*. 2020). Key VR design features, such as realistic environments, meaningful choices, interactivity, and immediate feedback, have been shown to support behavioural rehearsal and learning outcomes (Radianti *et al*. 2020).

However, the current evidence base remains limited in several important ways. Most studies rely on proxy outcome measures (e.g. attitudes, intentions, or knowledge), with few demonstrating sustained behavioural change. Moreover, higher-order cognitive outcomes such as critical thinking, problem-solving, or emotional regulation are rarely assessed, despite their relevance to long-term AOD prevention. Another gap lies in the limited integration of VR interventions into formal school curricula, reducing their educational relevance and scalability. Design improvements are also needed, particularly the use of compelling narratives, social interaction features (de la Rosa *et al*. 2023), and motivational reward mechanisms to deepen learning and engagement. When embedded in theory-driven frameworks, VR holds considerable promise as a scalable and emotionally engaging tool for adolescent AOD prevention (Durl *et al*. 2018, Mancuso *et al*. 2023). Yet, most existing interventions remain exploratory, with a narrow focus on short-term outcomes and limited insight into how immersive technologies can support deeper, transformative learning. These limitations underscore the need for more comprehensive, school-based evaluations that explore a broader range of cognitive, emotional, and behavioural impacts, particularly in light of the growing influence of digital media on young people’s health behaviours and decision-making.

In summary, although VR shows promise for adolescent AOD prevention, significant gaps remain regarding its influence on decision-making, peer resistance, and the transfer of skills beyond knowledge gains. In addition, its alignment with curriculum frameworks and its consistency with harm minimization and health literacy goals have not been fully examined. This study addresses these gaps by evaluating a third-iteration VR AOD intervention. As part of a broader school-based prevention programme, the experience reflects further advancements in narrative design, user engagement, and accessibility. Using a mixed-methods approach, the study explores how students and teachers perceive the intervention’s educational value, emotional resonance, and practical applicability, offering new insights into the role of VR in contemporary, digitally mediated health promotion.

Accordingly, this study examined a school-based VR intervention targeting adolescent AOD prevention. It addressed two research questions: (1) How does the VR experience support engagement, emotional involvement, and prevention-related skill development? (2) How do students and teachers perceive and experience a school-based VR intervention targeting AOD prevention?

## Materials and methods

**Figure 1:**
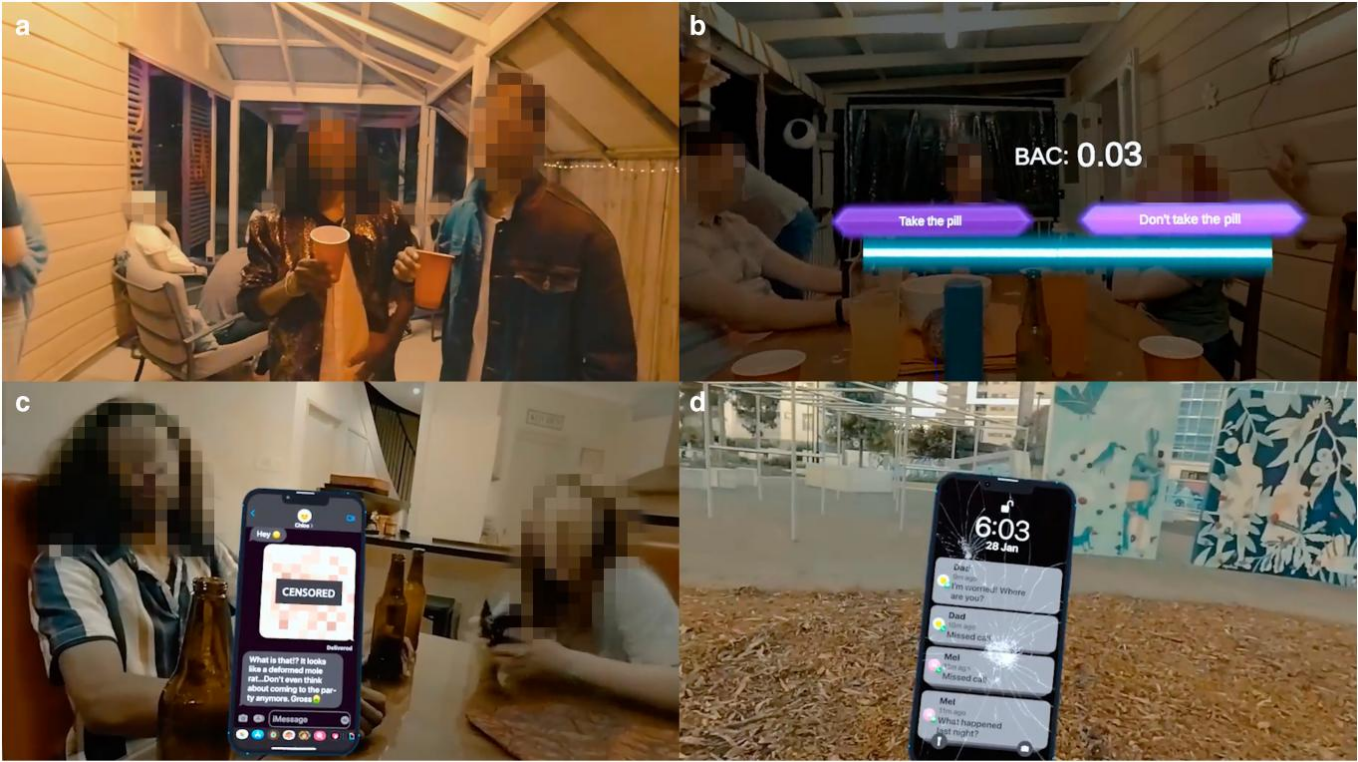
Representative screenshots of the VR environment. (a) Party scenario illustrating peer interaction and peer pressure cues. (b) Interactive decision point showing simulated blood alcohol concentration (BAC 0.03) feedback and branching choices. (c) In-game messaging simulating real-life digital communication. (d) Outcome screen depicting postevent consequences.

This study is part of a broader school-based VR AOD education initiative collaboratively developed by researchers and educators in Australia. The initiative integrates VR, gamified learning, and classroom-based discussion to engage secondary school students (Years 7–12; ∼12–17 years old), with a small number of 18-year-old students included in the sample due to grade-level variation. The VR intervention was codesigned with young people (Durl *et al*. 2017, Dietrich *et al*. 2019, Durl *et al*. 2022) and grounded in social cognitive theory, integrating behavioural science, psychology, game design, and VR development (see Fig. 1).

### Sample

A total of 277 students from four secondary schools in Australia participated in the VR intervention and completed an online postintervention survey. From this group, 124 students participated in 32 focus groups to provide deeper insights into their experience. Nine teachers who supervised the sessions were also interviewed. Student focus group participants were selected using a purposive sampling approach to capture diverse perspectives across year levels and schools.

### Data collection and procedure

The study was conducted in 1-hour classroom sessions with groups of ∼30 students. Each student used a VR headset to play the gamified simulation one to four times, depending on session duration and interest. After the experience, students completed an online survey measuring engagement, emotional responses, and skill development. Focus groups were conducted immediately following the survey to capture real-time reflections, while brief teacher interviews were conducted on-site or shortly after. This sequential approach supported the integration of experiential feedback with measurable outcomes.

### Measures

The survey included validated and adapted scales measuring experiential dimensions (engagement, immersion, and sensory fidelity), emotional responses, skill development (peer resistance, critical thinking, and problem-solving), and overall satisfaction. All items used Likert-type scales, with reliability confirmed via Cronbach’s alpha (see Table 1). Survey constructs were adapted from previous VR research including Dietrich *et al*. (2019), Weser *et al*. (2021), and Colombo *et al*. (2021).

**Table 1:** Reliability analysis.

Construct	*N* items	Cronbach’s alpha
*Engagement* ^a^	4^a^	0.728
*Sensory fidelity* ^a,b^	2^a^	0.619
	1^b^	
*Immersion* ^a^	3^a^	0.735
*Emotional responses* ^a^	5^a^	0.812
*Peer resistance and strategies* ^c^	3^c^	0.928
*Critical thinking and decision-*making^a^	4^a^	0.908
*Problem-solving skills* ^a^	4^a^	0.727

^a^ *Likert-scale 1 (strongly disagree) to 4 (strongly agree).*

^b^ *Likert-scale 1 (strongly agree) to 4 (strongly disagree).*

^c^ *Likert-scale 1 (strongly disagree) to 4 (strongly agree), 0 (I already had this skill).*

### Data analysis

#### Quantitative analysis

Survey data were analysed using SPSS (version 26.0). Descriptive statistics were used to summarize responses across experiential, emotional, and skill development domains. One-way analyses of variance (ANOVAs) were conducted to examine differences across gender groups on key outcomes including engagement, peer resistance strategies, and problem-solving skills.

#### Qualitative analysis

Focus group and interview transcripts were analysed in NVivo 14 using an inductive thematic approach. Open coding was conducted to identify recurring concepts, followed by axial and selective coding to organize and refine categories. A within-case and cross-case analysis enabled identification of commonalities and differences across participants. This process supported a rich understanding of student and teacher experiences with the intervention.

### Ethical approval

Ethical approval was obtained from the Griffith University Human Research Ethics Committee (GU 2021/003). Informed consent was obtained from all participants, and parental consent was secured for students under 18 years of age.

## Results

### Demographics

The study sample included 277 students from four schools in Australia. Students ranged in age from 12 to 18 years, with the highest proportion aged 14 (41.04%), followed by 15 (17.53%) and 13 (14.74%). Year-level distribution showed diverse representation across all stages of secondary schooling. The largest group was in Year 9 (48.80%), followed by Year 7 (13.60%), Year 10 (12.80%), Year 8 (12.40%), Year 11 (10.40%), and Year 12 (2.00%). This spread ensures that findings reflect the perspectives of students across junior, middle, and senior years. In terms of gender identity, 42.00% of participants identified as male, 55.60% as female, and 1.60% as nonbinary. An additional 0.80% preferred not to disclose. Regarding cultural background, 4.78% identified as Aboriginal, 1.20% as Torres Strait Islander, 1.59% as South Sea Islander, and 83.27% as non-Indigenous. A further 9.16% chose not to disclose.

### Behaviours

Self-reported behavioural data indicated that 45.53% of students had tried alcohol, 15.63% had tried marijuana, and 30.86% had vaped at least once. Among those who reported drinking alcohol, 19.84% consumed alcohol once a month, while smaller proportions drank two to three times per month (1.95%), weekly (2.33%), or more frequently (2.33%). Regarding marijuana use, 4.67% reported monthly use, 3.11% used it four or more times per week, and 1.56% used it two to three times weekly or two to four times monthly. For vaping, 10.89% were very occasional users, 2.72% vaped occasionally, and 8.17% reported frequent or often use. Peer pressure experiences were also explored: 21.74% had felt pressure to vape, 22.26% to drink alcohol, and 12.12% to use marijuana. These findings reinforce the relevance of peer-focused prevention education in adolescent populations.

### Quantitative results

#### Player experience

All participants completed the VR experience, but their level of engagement varied. The majority completed the simulation multiple times: 24.55% played twice, 23.10% played three times, and 38.99% engaged four or more times. Students exhibited diverse decision-making behaviours in the VR environment: 24.55% made thoughtful choices, 13.36% engaged in risk taking, 32.49% demonstrated mixed behaviour, 9.39% were impulsive, and 17.69% made random choices. This variation underscores individual differences in decision-making approaches, mirroring real-life complexities. Most students (57.04%) experienced no nausea or discomfort, while 25.27% reported mild, 11.55% moderate, and 6.14% severe discomfort. Additionally, 81.23% reported no technical issues, but 18.77% faced some difficulties. Regarding physical comfort, 71.48% of participants felt comfortable using the headset, though a notable 28.52% reported discomfort, indicating a need for future improvements in VR usability.

The reliability and descriptive statistics for experiential dimensions, skill development, and emotional responses are presented in Table 2. This table provides an overview of the key experiential and outcome measures, which are further elaborated in the following sections.

**Table 2:** Experiential dimensions, skill development, and emotional responses.

*Measure*	*N*	*Mean*	*SD*	*Source*
***Engagement*** ^a^	274	**2**.**480**	0.598	(Weser *et al*. 2021)
*1. I felt connected to the other characters in the game*	274	2.215	0.808	
*2. I felt as if I was in a real place during the Blurred Minds VR experience*	274	2.701	0.815	
*3. I was not aware of my real environment*	274	2.401	0.864	
*4. I was completely captivated by the virtual world*	274	2.602	0.842	
***Sensory fidelity*** ^a,b^	273	**2**.**383**	0.623	(Weser *et al*. 2021)
*1. I felt connected to my character in the game* ^a^	273	2.315	0.905	
*2. My experience in the virtual environment seemed like a real-world experience* ^a^	273	2.491	0.871	
*3. I felt like I was just seeing pictures or a movie, rather than visiting a place* ^b^	273	2.344	0.799	
***Immersion*** ^a^	271	**2**.**784**	0.584	(Weser *et al*. 2021)
*1. I liked the way the game looked*	271	2.675	0.719	
*2. The sounds I heard in the game helped me to feel like I was visiting a real place*	271	2.849	0.717	
*3. I felt that the virtual world was all around me*	271	2.827	0.732	
***Presence*** ^a^	269	**2**.**680**	0.618	(Weser *et al*. 2021)
*I felt present in the virtual space*	269	2.680	0.618	
***Emotional responses*** ^a^	267	**1**.**918**	0.585	(Skorka-Brown *et al*. 2015; Chen *et al*. 2021; Colombo *et al*. 2021, Primavera *et al.*, 2024)
*1. I felt an increase in craving after seeing drugs in the game*	267	1.712	0.801	
*2. My anxiety increased as I navigated through the VR experience*	267	1.974	0.782	
*3. I experienced a strong emotional response when faced with drug-related scenarios in VR*	267	2.030	0.827	
*4. The virtual environment made me feel tense and triggered an emotional reaction (e.g. unease)*	267	1.955	0.724	
*5. I found it difficult to regulate my emotions while immersed in the VR scenario*	266	1.921	0.746	
***Peer resistance and refusal strategies*** ^c^	194	**3**.**011**	1.291	(Dietrich *et al*. 2019, Durl *et al.* 2020)
*1. After the VR game, do you feel equipped to resist peer pressure involving vapes?*	185	2.984	1.349	
*2. After the VR game, do you feel equipped to resist peer pressure involving alcohol?*	189	2.984	1.362	
*3. After the VR game, do you feel equipped to resist peer pressure involving marijuana?*	190	3.074	1.367	
***Critical thinking and decision-making*** ^a^	260	**2**.**863**	0.732	(Dietrich *et al*. 2019, Weser *et al*. 2021)
*1. Evaluate pros and cons of substances before making a decision*	260	2.915	0.839	
*2. Question information before making a decision*	260	2.869	0.800	
*3. Make decisions based on evidence, not intuition*	260	2.765	0.858	
*4. Consider all possible outcomes before making a decision*	260	2.904	0.876	
***Problem-solving skills*** ^a^	257	**2**.**791**	0.694	(Dietrich *et al*. 2019, Weser *et al*. 2021)
*1. Think clearly when faced with tough social decisions*	257	2.782	0.814	
*2. Better evaluate options*	257	2.809	0.790	
*3. Anticipate consequences of actions*	257	2.852	0.801	
*4. Solve problems under pressure*	257	2.720	0.824	
***Satisfaction*** ^d^	253	**2**.**846**	0.681	(Dietrich *et al*. 2019)
*How satisfied were you with the VR experience?*	253	2.846	0.681	

Bold values indicate the mean scores at the construct (scale) level for each experiential dimension, while non-bold values represent item-level scores.

^a^ *Likert-scale 1 (strongly disagree) to 4 (strongly agree).*

^b^ *Likert-scale 1 (strongly agree) to 4 (strongly disagree).*

^c^ *Likert-scale 1 (strongly disagree) to 4 (strongly agree), 0 (I already had this skill).*

^d^Likert-scale 1 (very dissatisfied) to 4 (very satisfied).

#### Experiential dimensions and presence

Students reported positive experiences in several areas of immersion and environmental design. Immersion received a high mean score (M = 2.784; SD = 0.585), supported by positive feedback on the game’s visual design (M = 2.675; SD = 0.719) and spatial sound design (M = 2.849; SD = 0.717). Many students felt surrounded by the virtual environment (M = 2.827; SD = 0.732), reinforcing the simulation’s capacity to create a believable immersive context. Presence in the virtual space was rated as moderate (M = 2.680; SD = 0.618), suggesting that while many students felt immersed, their sense of being ‘in’ the virtual environment was somewhat varied. Engagement (M = 2.480; SD = 0.598) and sensory fidelity (M = 2.383; SD = 0.623) were rated moderately, indicating room for future refinement in areas such as character connection and realism. While students interacted positively with the environment, the emotional connection to avatars and depth of perceived realism could be strengthened.

#### Skill development

Participants showed positive outcomes in several skill development domains. Critical thinking and decision-making achieved one of the highest composite scores (M = 2.863; SD = 0.732). Students reported that the game encouraged them to evaluate the pros and cons of substance use (M = 2.915), question information before acting (M = 2.869), and consider the consequences of their choices (M = 2.904). These results indicate that the VR experience contributed meaningfully to reflective thinking. Problem-solving skills also performed well (M = 2.791; SD = 0.694). Students reported improved capacity to think clearly in complex social situations (M = 2.782), evaluate decision-making options (M = 2.809), and anticipate the outcomes of their actions (M = 2.852). These findings highlight the intervention’s role in equipping students with practical, transferable decision-making tools. Peer resistance and refusal strategies (M = 3.011; SD = 1.291) were rated very high, suggesting that the experience may have helped students feel more equipped to manage peer pressure related to alcohol, vaping, and marijuana.

#### Emotional responses and satisfaction

Emotional responses were measured to assess students’ regulation and psychological comfort during the experience. Scores were generally low (M = 1.918; SD = 0.585), with students reporting manageable levels of anxiety (M = 1.974), suggesting that the intervention encouraged reflection without causing excessive distress. Craving responses were also minimal (M = 1.712; SD = 0.801), reinforcing the appropriateness of the content for a school setting. Satisfaction with the VR intervention was positive (M = 2.846; SD = 0.681), indicating that students valued the format and considered it a relevant and engaging educational tool. Overall, the response profile suggests an intervention that was well received and aligned with experiential learning goals in school-based AOD education.

#### Gender differences

A one-way ANOVA (Supplementary File 1) was conducted to examine gender-based differences across key outcome variables. The analysis included male (*n* = 105), female (*n* = 139), nonbinary (*n* = 4), and students who preferred not to disclose their gender (*n* = 2). Significant differences were observed in three domains: engagement, peer resistance strategies, and problem-solving skills. Female students reported significantly higher engagement (M = 2.624; SD = 0.521) than male students [M = 2.288; SD = 0.594; *F* (3, 246) = 8.142; *P* < .001]. This suggests that female participants may have found the VR simulation more immersive or personally relevant. For peer resistance strategies, males reported higher mean scores (M = 3.242; SD = 1.371) than females [M = 2.800; SD = 1.159; *F* (3, 186) = 2.785; *P* = .042], indicating possible differences in perceived preparedness to resist peer pressure. Significant differences were also found in problem-solving skills [*F* (3, 186) = 3.270; *P* = .022], with nonbinary participants reporting the highest scores (M = 3.500; SD = 0.577), followed by females, males, and undisclosed participants. No significant gender differences were found for immersion, sensory fidelity, emotional responses, presence, satisfaction, substance use behaviours, or critical thinking. These results suggest that while some variation exists across gender in specific areas, the intervention generally provided a consistent experience for most participants.

### Qualitative results

#### Thematic analysis of student insights

A total of 124 high school students participated in 32 focus groups across six dates and locations. The thematic analysis of student focus groups revealed rich and varied perspectives on their engagement with the VR intervention (summarized in Supplementary File 2). Four overarching themes emerged: *virtual reality content and narrative, immersion and realism, educational value and real-world relevance, and suggested improvements.* These themes provide insight into the emotional, cognitive, and practical dimensions of student engagement with the virtual simulation.

##### Virtual reality content and narrative

Students described the storyline as compelling and emotionally impactful, with many citing specific scenes that left lasting impressions, such as party environments, emergency situations, and peer interactions. The branching structure of the narrative allowed students to explore consequences based on their decisions, which helped to personalize the experience. While many found the content engaging and reflective of real-life situations, some questioned the lack of complexity in the storyline or noted areas where character development and dialogue could be improved for greater relatability. One student reflected, ‘It felt like I was in a real situation where I had to think about what would happen next’. Another added, ‘The ambulance scene stuck with me—it showed what can actually happen when things go wrong at parties’.

##### Immersion and realism

The sense of immersion varied across students. Several participants reported feeling ‘inside the moment’ or described the experience as emotionally vivid, especially during scenarios that involved peer pressure or risk. The VR environment’s visual and auditory elements supported this immersion, although limitations in graphic quality, character animation, and headset functionality occasionally disrupted it. A few students reported physical discomfort or technical issues that affected their engagement with the simulation. One participant noted, ‘I kind of forgot I was in a classroom—it really pulled me in’. In contrast, another reflected, ‘It felt real at times, but then something like a glitch or weird movement would snap you out of it’.

##### Educational value and real-world relevance

Students consistently identified the intervention as a meaningful learning experience. Many reflected on how the VR format made them more aware of the social dynamics surrounding substance use and the potential consequences of different decisions. They appreciated being able to practice refusal skills and see outcomes play out in a simulated environment, which helped to reinforce messages about self-efficacy and personal agency. Several students noted that the intervention helped them think about how they might respond in future real-world scenarios. One remarked, ‘It shows how to deal with pressure in the moment, which is hard to explain in a classroom’. Another shared, ‘It made me realize you can actually walk away, and that’s okay’.

##### Suggested improvements

While overall feedback was positive, students shared detailed suggestions for enhancing the experience. Some recommended expanding interaction options, allowing for more freedom to explore environments or make nuanced decisions. Others expressed a desire for improved character realism, better graphics, or more accurate audio syncing. Content suggestions included more culturally diverse characters, different types of social settings, and broader consequences beyond immediate peer reactions. A small number of students also suggested extending the gameplay duration or incorporating multiple episodes to deepen the learning experience. One student shared, ‘I think having characters from different backgrounds or schools would help more people relate to it’. Another suggested, ‘If there were different endings depending on your choices, it would feel more real’.

#### Thematic analysis of teacher insights

A total of nine high school teachers participated in nine interviews across six dates and locations. The key themes and subthemes identified from teacher interviews are shown in Supplementary File 3.

##### Immersion and realism

Teachers generally agreed that the VR experience created a realistic social environment, particularly through familiar elements such as the text message interaction feature. One teacher remarked, ‘The text message interaction is very realistic and aligns perfectly with how students communicate in real life’. This design element was considered helpful in supporting immersion and encouraging natural student engagement with the scenarios. Several teachers noted that the environmental details, such as bedrooms and party scenes, closely resembled typical adolescent contexts. One teacher observed, ‘Looking around, everything looked like a teenage bedroom or a teenage party or choices and decisions that you would have to make as a teenager’. However, some teachers questioned the relatability of specific scenes, such as discovering alcohol in a parent’s bedroom, and suggested incorporating more relevant settings such as school environments, public places, or social media interactions to better reflect the range of spaces where young people encounter substance-related decisions. Another participant commented on the sensory realism of the experience, stating, ‘It was amazing! I couldn’t believe how realistic it was. Honestly, I felt a little dizzy—like I might fall over at some point’.

##### Decision-making and educational value

Teachers identified the VR intervention as a valuable educational tool for supporting student decision-making, particularly in the context of alcohol- and drug-related scenarios. They noted that the branching pathways and visible consequences of each choice allowed students to engage with content in a way that felt personal, reflective, and relevant. Several educators remarked that this approach made the learning more meaningful than traditional classroom delivery. One teacher explained, ‘They can replay it with different decisions. It may have a bigger impact than me just standing up there’. The opportunity to simulate risk-related decision-making in a safe environment was frequently highlighted as a strength. Teachers commented that the VR format enabled students to ‘experience’ the consequences of their actions without real-world harm. This design was seen as especially important for younger students who may not have encountered such situations before. As one teacher noted, ‘They get to see what might happen before it actually happens in real life’. Educators also observed that the experience encouraged reflection and discussion beyond the headset, suggesting a spill-over effect into broader learning. One participant shared, ‘It made them talk—like, they wanted to debrief. They were asking each other, ‘What did you pick? What happened to you?’ This social learning aspect was seen as enhancing the educational impact, reinforcing that students weren’t just passively consuming content but actively negotiating their understanding of peer influence and consequences. Moreover, several teachers viewed the intervention as aligning with broader goals of promoting agency and refusal skills. They described how students were prompted to consider not only what choices to make, but why those choices mattered. One teacher commented, ‘It teaches them to stop and think—something we try to do in health but don’t always have the tools for’. Another added, ‘It shows the chain reaction—how one decision can lead to more pressure, or something serious’.

##### Applicability and curriculum fit

Teachers spoke positively about the intervention’s relevance across different age groups and its potential for integration within various areas of the curriculum. Several educators identified Years 7–9 (∼12–15 years old) as the most appropriate target group, highlighting the alignment between the simulation’s content and the developmental needs of early secondary students. Health and wellbeing subjects were most frequently mentioned as a natural fit, though teachers also saw potential to embed the experience in broader discussions about respectful relationships, peer dynamics, and risk education. A teacher reflected, ‘It ties in well with what we’re already doing in health—it’s just a better way of doing it’. Others highlighted that the scenarios could support pastoral care and behaviour-focused programmes, particularly when used as a stimulus for group discussion and classroom reflection.

##### Suggested improvements

While teachers responded positively overall, they offered several constructive suggestions to enhance the VR experience’s educational value and classroom integration. A common recommendation was to expand the range and complexity of decision-making pathways to allow for more nuanced choices and varied outcomes. Some teachers noted that the current options could feel limited, reducing opportunities for deeper reflection. Others proposed that including branching consequences tied to long-term outcomes—such as relationships, health, or school impact—could make scenarios more impactful. Teachers also suggested broadening the diversity of characters and cultural contexts to improve relatability for a wider range of students. Technical refinements were highlighted as well, including improving sound clarity, making text easier to read, and ensuring smoother transitions between scenes. One teacher explained, ‘The game ended a bit too quickly both times, so having more choices and outcomes would improve it’. A few teachers also recommended more scaffolding to support classroom debriefing, such as a companion guide or postgame prompts to help facilitate structured discussion. As one teacher shared, ‘It would help to have some discussion starters or reflection questions to tie it back to the health curriculum’.

## Discussion

This study contributes to the growing field of immersive technologies in health promotion by examining how VR can support adolescent learning within the context of alcohol, vaping, and other drug prevention. Drawing on both quantitative and qualitative insights from students and teachers, the findings offer valuable guidance for the design and implementation of VR-based interventions in school-based health education. Importantly, this research builds on and extends the existing evidence base by exploring how VR can influence a wide range of psychological, emotional, and behavioural factors such as engagement, immersion, sensory fidelity, emotional responses, peer resistance, critical thinking, problem-solving, and overall satisfaction. The findings from this study highlight several key themes that can inform the future development and implementation of VR interventions for adolescent AOD prevention. Drawing on both quantitative outcomes and rich qualitative feedback, four core areas emerged: (i) the role of immersion and realism in fostering engagement, (ii) the importance of reflective and experiential learning, (iii) the need for curriculum alignment and practical implementation strategies, and (iv) the critical importance of inclusive, youth-centred design. The following sections unpack each of these themes, beginning with how immersion and realism shape student engagement in VR-based health education.

### Engagement through immersion and realism

Immersive and contextually relevant VR experiences appear well positioned to enhance user engagement, particularly among young people. Both students and teachers in this study recognized that sensory fidelity, relatable environments, and familiar interaction formats (such as messaging interfaces) contributed to students’ psychological immersion and perceived realism. These features are consistent with literature identifying immersion as a catalyst for behavioural engagement and presence (Hennig-Thurau *et al*. 2025), which can support deeper learning and emotional processing in digital health interventions (Creed *et al*. 2024). To optimize immersion, future VR interventions should prioritize user-centred design, ensuring that characters, environments, and scenarios reflect the lived experiences and communication styles of the target population (Creed *et al*. 2024). Integrating realistic social contexts (e.g. schools, social media, or public events) can make health scenarios feel more applicable to daily life, strengthening relevance and engagement. VR scenarios that combine sensory fidelity and narrative immersion can support metacognitive awareness and emotional regulation (Mitsea *et al*. 2025). Moreover, given the dual potential of digital environments to both promote and harm health (Holly *et al*. 2025), developers must carefully balance immersive appeal with evidence-based health messaging. These immersive design elements capture attention and lay the groundwork for deeper learning particularly when paired with opportunities for reflection and decision-making within the experience. The next theme explores how VR can promote reflective and experiential learning to support skill development and behavioural change.

### Promoting reflective and experiential learning

Health education VR interventions may improve content knowledge, engagement, and motivation especially when gamified and personalized (Marougkas *et al*. 2024). Specifically, VR’s interactive format offers an opportunity to model and rehearse health-related decision-making in a safe, simulated environment (Jiang *et al*. 2026). Interventions that allow users to explore consequences, via branching narratives or feedback mechanisms, may be particularly effective for adolescents, who are developing critical thinking, emotion regulation, and peer negotiation skills (Skinner *et al*. 2024). Experiential learning theory supports this approach, suggesting that behaviour change is more likely when learners actively engage in reflection and simulation (Kolb 2014). Furthermore, immersive storytelling and emotionally charged scenarios can help users internalize key messages and prepare for real-life challenges (Goldsmith *et al*. 2023). Emotional experiences in VR, whether positive or negative, are also more vividly remembered than neutral ones, highlighting the powerful role of affect in learning and retention (Mancuso *et al*. 2023). Previous studies on digital health tools also show promise for promoting self-efficacy and intention to change behaviour among young people (Nyman *et al*. 2022). VR, by enabling lived simulation of peer pressure or risk situations, appears uniquely positioned to bridge knowledge and practice in adolescent health promotion. Digital interventions also offer less stigmatizing and more engaging options for youth substance use prevention and early intervention (Monarque *et al*. 2023). However, it remains important to ensure such interventions cater to diverse learners, including those with sensory sensitivities or differing levels of digital literacy. As Creed *et al*. (2024) emphasize, inclusivity in VR design is vital for equitable access and learning outcomes, and future interventions must continue to prioritize user-centred design principles across varying student needs. While VR holds strong potential for enhancing self-efficacy and decision-making through lived simulation, its impact is amplified when embedded meaningfully within broader educational frameworks. The following section examines how curriculum alignment and practical implementation strategies can support the sustainable integration of VR in school-based health promotion.

### Curriculum alignment and implementation considerations

For VR interventions to have sustainable impact in school-based health promotion, alignment with curriculum standards and practical classroom requirements is critical. Educators in this study reported that immersive tools can complement traditional health and wellbeing curricula by providing engaging, context-rich scenarios for skill building and values exploration. This is consistent with previous literature suggesting that integrating digital tools into school programmes can enhance student motivation and deepen learning when linked to clear learning objectives and outcomes (AlGerafi *et al*. 2023). It also supports calls for school-based health promotion to be both evidence informed and contextually responsive (DiCasmirro *et al*. 2025, Raeside 2025).

To support implementation, future VR initiatives should consider integrating structured teaching materials, such as lesson plans, discussion prompts, and educator guides, that facilitate reflection and discussion beyond the headset, to support knowledge transfer. As noted in studies on health information visualization in VR, scaffolding learning through appropriate support materials can significantly improve comprehension and behaviour-related outcomes (Ferguson *et al*. 2024). These materials can enhance knowledge transfer and assist teachers in linking virtual experiences with classroom learning goals. Effective implementation also depends on ensuring that VR tools are accessible, relevant, and adaptable to the diverse realities of the classroom. The next theme considers how inclusive design and equity-focused practices are essential for maximizing impact and preventing the deepening of existing digital and health disparities.

### Design and equity considerations for youth-focused virtual reality

The success of VR in health promotion relies not only on its technical quality but also on its cultural relevance, inclusivity, and accessibility as highlighted in our study. Participants in this study emphasized the importance of including diverse characters and adaptable content to ensure interventions are relatable across different student groups. For VR to serve all students, content must reflect diverse backgrounds and offer adaptable features. While standalone VR headsets are becoming more affordable, disparities in access to technology can exacerbate educational and health inequities. Research stresses the importance of participatory design practices that engage youth and account for a variety of learning needs (Creed *et al*. 2024). As the technology evolves, inclusive VR design will require attention to accessibility features, user agency, and equitable hardware access in school settings (Creed *et al*. 2024). This includes the development of disability-aware systems, customizable avatars and interfaces, and inclusive character representation. Addressing these aspects from the outset is essential to prevent the exacerbation of digital inequalities, particularly in public education contexts where access and cultural relevance must be prioritized (Creed *et al*. 2024). Health promotion frameworks must also account for digital determinants of health, recognizing how digital access, literacy, and algorithmic systems shape equity and wellbeing (Holly *et al*. 2025).

### Implications for future research and practice

The findings from this evaluation reinforce the potential of VR as a tool for adolescent health promotion, particularly when interventions are grounded in realistic, socially situated scenarios. For practice, this suggests that health education programmes could benefit from incorporating immersive components that allow students to actively engage with behavioural content, rather than passively receiving information. When aligned with curriculum frameworks and supported by teacher-facing materials, VR may enhance the delivery of complex topics such as substance use, peer pressure, and refusal strategies in age-appropriate and engaging ways (Burnett *et al*. 2023, Raeside 2025). For researchers, these results highlight the need for more rigorous evaluation of VR interventions, particularly in capturing behavioural, cognitive, and emotional outcomes alongside engagement metrics. Despite promising preliminary results, the evidence base for VR in health promotion remains limited, with most existing interventions supported by only basic evaluations. This study contributes to addressing that gap by offering a mixed-methods exploration of student and teacher experiences. However, more comprehensive and longitudinal research is required to assess sustained impact and behavioural change. Future studies should compare VR-based approaches with traditional and other digital interventions to determine their unique contributions to learning and prevention outcomes (DiCasmirro *et al*. 2025). Advancing this field will require interdisciplinary collaboration between educators, technologists, behavioural scientists, and public health practitioners to ensure VR tools are pedagogically sound, evidence informed, and practically scalable across diverse school and community settings (Raeside 2025). Combining immersive technologies with adaptive learning systems and AI may further enhance engagement and personalization for diverse learners (Mitsea *et al*. 2025).

### Limitations

This study was limited by its post-only survey design, which prevents direct assessment of changes in student outcomes attributable to the intervention. Without baseline data, it is not possible to determine the extent to which the VR experience influenced knowledge, attitudes, or intended behaviours. Due to time constraints in schools and the brief workshop format of the VR intervention, preintervention data were not collected. Consequently, this study focused on students’ engagement and perceptions of the VR experience rather than pre–postbehavioural change assessment. Additionally, the reliance on self-reported measures introduces the potential for response bias, including social desirability effects. The qualitative feedback, while rich, was based on a convenience sample and may not reflect the full diversity of student and teacher perspectives. These limitations suggest caution in generalizing findings beyond the study context. Future research employing pre–post or control group designs will be critical to strengthen the evidence base for VR interventions in health promotion. Future research should also prioritize longitudinal evaluation, diverse school settings, and inclusive implementation to ensure equitable access and sustained impact.

## Conclusion

This study adds to the growing body of evidence on the role of immersive technologies in adolescent health promotion by evaluating a third-iteration VR intervention targeting alcohol, vaping, and drug prevention in Australian schools. It builds on and extends the existing evidence base by exploring how VR can influence a wide range of psychological, emotional, and behavioural factors such as engagement, immersion, sensory fidelity, emotional responses, and peer resistance while also supporting the development of critical decision-making and refusal skills. Findings demonstrate that immersive, gamified experiences can meaningfully engage students and align with harm minimization principles and curriculum goals. By integrating youth perspectives and educator insights, the study reinforces the importance of user-centred design and contextual fit in school-based interventions. While opportunities remain for technical and narrative refinement, the intervention was positively received and considered educationally valuable by both students and teachers. Importantly, it offers a scalable, curriculum-compatible tool for supporting skill rehearsal and reflective learning in emotionally engaging, lower-stakes environments. As schools seek innovative ways to address substance use, VR stands out as a promising and adaptable platform.

## Supplementary Material

daag002_Supplementary_Data

## Data Availability

The datasets generated and analysed during the current study are not publicly available due to ethical restrictions, but are available from the corresponding author on reasonable request.
