# Supplementary material for: Virtual reality in school-based health promotion: a mixed-methods evaluation of adolescent alcohol, vaping, and other drug use prevention
Source: Health Promot Int. 2026 Jan 27;41(1):daag002. doi: 10.1093/heapro/daag002 (PMC12835819; doi:10.1093/heapro/daag002)
Supplement: daag002_Supplementary_Data [file daag002_supplementary_data.doc]

**Supplementary files**

***Supplementary file 1. Gender difference analysis***

| **ANOVA** | | | | | | |
| --- | --- | --- | --- | --- | --- | --- |
|  | What is your gender identity? (Mean ± SD) | | | | F | p |
|  | Male(n=105) | Female(n=139) | Non-binary(n=4) | Prefer not to say(n=2) |  |  |
| **Player Experience** | 3.229±0.753 | 3.058±0.662 | 3.000±0.408 | 3.000±0.707 | 1.258 | 0.289 |
| **Engagement** | 2.288±0.594 | 2.624±0.521 | 2.000±0.913 | 2.500±0.707 | 8.142 | 0.000** |
| **Sensory Fidelity** | 2.298±0.642 | 2.456±0.603 | 2.167±0.793 | 1.667±0.471 | 2.319 | 0.076 |
| **Immersion** | 2.743±0.548 | 2.836±0.453 | 2.500±1.061 | 2.625±0.177 | 1.202 | 0.310 |
| **Presence** | 2.610±0.700 | 2.755±0.523 | 2.250±0.500 | 2.500±0.707 | 1.926 | 0.126 |
| **Emotional Responses** | 1.886±0.643 | 1.921±0.535 | 1.950±0.681 | 1.600±0.849 | 0.260 | 0.854 |
| **Peer Resistance & Strategies** | 3.242±1.371 | 2.800±1.159 | 3.333±1.528 | 4.500±0.707 | 2.785 | 0.042* |
| **Critical Thinking and Decision** | 2.786±0.864 | 2.915±0.607 | 3.500±0.408 | 2.375±0.884 | 1.963 | 0.120 |
| **Problem-Solving Skills** | 2.664±0.796 | 2.856±0.594 | 3.500±0.577 | 2.375±0.177 | 3.270 | 0.022* |
| **Substance Use Behaviours** | 4.533±0.809 | 4.609±0.737 | 4.000±2.000 | 4.833±0.236 | 0.948 | 0.418 |
| **Satisfaction** | 2.800±0.656 | 2.899±0.684 | 2.500±1.000 | 2.500±0.707 | 0.969 | 0.408 |
| * p<0.05 ** p<0.01 | | | | | | |

***Supplementary file 2. Thematic analysis of student insights***

| **Theme** | **Definition** | **Specific themes** | **Definition** |
| --- | --- | --- | --- |
| **VR content and narrative (142)** | The core narrative, settings, and character activities that shape the overall VR game experience. | **Scenes in the game (41)** | The virtual environments and backgrounds that create immersion. |
|  |  | **Character behaviours and interactions (55)** | The actions and interactions of characters influencing player engagement. |
|  |  | **Choice tendency (46)** | The decisions and preferences made by players within the game. |
| **Immersion and realism (213)** | The player’s subjective perception and evaluation of realism and immersion in the VR environment. | **Realism and immersion experience (81)** | Players’ overall experience of realism and immersion, including their perceptions of authenticity as well as the design and technical factors that shape their sense of presence. |
|  |  | **Memorable Moments (30)** | \|  \| \| --- \|  \| Key moments in the game that leave a strong impression on players. \| \| --- \| |
|  |  | **Attitude towards VR (22)** | Players’ overall opinions and feelings about VR technology. |
|  |  | **Overall evaluation of the experience (42)** | A comprehensive assessment of the entire game experience by players. |
|  |  | **Satisfaction (38)** | The degree to which players are content with the game and its quality. |
| **Educational value and real-world relevance (153)** | The potential educational benefits and real-world behavioural influences of the game. | **Educational and warning significance (34)** | The game’s role in delivering educational messages and warnings. |
|  |  | **Impact on real - life behaviours (31)** | Changes or adaptations in players’ real-world behaviour after playing. |
|  |  | **Choices and decisions faced (88)** | Important decisions players must make in-game and in real life. |
| **Suggested improvements**  **(89)** | Players’ opinions and expectations for future enhancements of the game. | **Improvement of content and plots (21)** | Recommendations for enriching storylines and game content. |
|  |  | **Game problem feedback (33)** | Critical feedback regarding functional, visual, and narrative shortcomings that hinder gameplay quality and immersion. |
|  |  | **Optimisation of technology and equipment (18)** | Suggestions for improving hardware and software performance. |
|  |  | **Enhancement of game functions (17)** | Requests for adding or improving game features and gameplay. |

***Supplementary file 3. Thematic analysis of teacher’s insights***

| **Theme** | **Sub-themes** | **Count** |
| --- | --- | --- |
| Immersion and realism | Environmental fidelity, detail realism, message integration | 9 |
| Decision-making and educational value | Consequences, critical thinking, discussion prompting, behavioural reflection | 22 |
| Applicability and curriculum fit | Age adaptability, subject fit, social topic relevance, student relatability | 20 |
| Suggested improvements | Choice depth, technical fixes, scene updates, knowledge content, equipment | 13 |
